# Seminal vesicle interfraction displacement and margins in image guided radiotherapy for prostate cancer

**DOI:** 10.1186/1748-717X-7-139

**Published:** 2012-08-13

**Authors:** Daisy Mak, Suki Gill, Roxby Paul, Alison Stillie, Annette Haworth, Tomas Kron, Jim Cramb, Kellie Knight, Jessica Thomas, Gillian Duchesne, Farshad Foroudi

**Affiliations:** 1 Radiation Oncology Division, Peter MacCallum Cancer Centre, Locked Bag 1, A'Beckett Street, East Melbourne, VIC, 8006, Australia; 2 Physical Sciences Department, Peter MacCallum Cancer Centre, East Melbourne, VIC, Australia; 3 Radiation Therapy Services, Peter MacCallum Cancer Centre, East Melbourne, VIC, Australia; 4 Department of Pathology, The University of Melbourne, East Melbourne, VIC, Australia

**Keywords:** Seminal vesicle, Interfraction displacement, Margins, Prostate cancer, Radiotherapy

## Abstract

**Background:**

To analyze interfraction motion of seminal vesicles (SV), and its motion relative to rectal and bladder filling.

**Methods and Materials:**

SV and prostate were contoured on 771 daily computed tomography “on rails” scans from 24 prostate cancer patients undergoing radiotherapy. Random and systematic errors for SV centroid displacement were measured relative to the prostate centroid. Margins required for complete geometric coverage of SV were determined using isotropic expansion of reference contours. SV motion relative to rectum and bladder was determined.

**Results:**

Systematic error for the SV was 1.9 mm left-right (LR), 2.9 mm anterior-posterior (AP) and 3.6 mm superior-inferior (SI). Random error was 1.4 mm (LR), 2.7 mm (AP) and 2.1 mm (SI). 10 mm margins covered the entire left SV and right SV on at least 90% of fractions in 50% and 33% of patients and 15 mm margins covered 88% and 79% respectively. SV AP movement correlated with movement of the most posterior point of the bladder (mean R^2^ = 0.46, SD = 0.24) and rectal area (mean R^2^ = 0.38, SD = 0.21).

**Conclusions:**

Considerable interfraction displacement of SV was observed in this cohort of patients. Bladder and rectal parameters correlated with SV movement.

## Background

In prostate cancer radiotherapy, proximal seminal vesicles (SV) are usually included in the clinical target volume (CTV) in intermediate to high risk prostate cancer. Bayman *et al.* conducted a review of prostatectomy series and revealed that the risk of SV invasion in intermediate risk patients is between 13 and 22% [1]. As a result, recommendations were made to encompass SV in patients with at least one risk factor of PSA >10, Gleason Score ≥7, T stage >2A, or percentage of positive biopsy >50%. Due to its proximity to rectum and bladder, SV inclusion may result in an increased rectal or bladder toxicity [2]. Methods which reduce SV CTV to planning target volume (PTV) margins may reduce side effects from radiotherapy.

Image guided radiotherapy (IGRT) is increasingly being used in the treatment of prostate cancer [3]. In IGRT, the prostate gland is localized before each fraction of treatment to allow for the correction of prostate displacement. This is usually performed without localization of the SV because SV are usually not visible on planar imaging and can be difficult to identify on cone beam computed tomography (CT).

Internal organ movement of the prostate is well documented in the literature [4]. SV motion has been relatively less well described, and is summarized in Table 1. We aimed to use daily CT on rails images to i) quantify the inter-fraction SV displacement in relation to the centroid of prostate during a course of radiotherapy for prostate cancer, simulating IGRT targeting the prostate with translations (but not rotations), ii) assess geometry-based (rather than dose-based) margins required to completely cover the SV for any percentage of fractions, using isotropic expansion of reference contours to allow for both movement and deformation independent of treatment technique and iii) evaluate correlations between SV motion and changes in the rectum and bladder, two structures which may be easier to see than the SV themselves using soft tissue IGRT.

**Table 1:** Studies analysing seminal vesicle motion and margins

**Authors**	Movements LR/AP/SI* (mm)	**Margins**	**Localisation†**	**Method for margins**	**Deformation**	**Patients**	**Scans per patient**
Current work	Centre of volume: Systematic 1.9/2.9/3.6. Random 1.4/2.7/2.1	Geometric method approx 15 mm (whole SV and inf 2.5 cm). Van Herk Formula approx 10 mm.	Prostate centroid	Geometrical expansion of reference SV contours, and Van Herk Formula	Yes	24	32
Frank *et al.*[7]	Centre of volume: Systematic 1.9/7.3/4.5. Random 0.4/1.2/0.6	≥ 10 mm 10 mm covers SV AP variation in 86% of treatments	Pubic symphysis	Geometrical	No	15	24
AHerne *et al.*[8]	N/A	5 mm cover both SVs in 56.2% of fractions,10 mm cover 95.5%, 15 mm cover all	Prostate fiiducial markers	Geometrical - margin to cover fiducial marker in SV	No	9	8-11
van der Wielen *et al.*[14]	Deformation + movement approx 3.0 (1.7 in LR)	Approx 9–10 mm (illustrative).	Prostate fiducial markers	Van Herk Formula (illustrative)	Yes	21	4
Mutanga *et al.*[15]	Used data above	8 mm margin insufficient (IMRT)	Prostate fiducial markers	Dose (simulation including deformation with deformable registration)	Yes	21	4
Liang *et al.*[16]	Maximize overlap of SV volume. Systematic 1.1/2.9/2.2. Random 1.2/2.4/1.9	Minimum margins 4.5 mm (IMRT)	Prostate (maximize overlap of 3D volume)	Dose (deformable registration)	Yes	24	16
O’Daniel *et al.*[17]	N/A	Study compared alignment technique and found 5 mm margins give a minimum of 92% of dose to SV using IMRT	Prostate centroid	Minimum dose in any fraction	Yes	10	24
Meijer *et al.*[18]	N/A	8 mm (inferior 2 cm of SV) (IMRT)	Prostate fiducial markers	Dose (deformable registration)	Yes	30	8
Smitsmans *et al.*[19]	Image based registration of central (sup/inf) part of SV Systematic 1.6/2.8/-- Random 2.0/3.1/--	4.6 mm LR 7.6 mm AP	Prostate fiducial markers	Van Herk Formula	No	13	23

*AP = anterior/posterior, LR = left/right, SI = superior/inferior† Using translations alone.

## Methods and materials

### Patient demographics

With human research ethical board approval, CT datasets of 24 consecutive patients treated radically for prostate cancer with daily pre-treatment CT on rails imaging were evaluated. Patient enrollment and treatment setup have been documented in our previous publication [5].

A total of 771 scans were obtained between the 24 patients (median 32 scans each, range 18 – 36). Median age was 72 years (range 55 – 78 years). Eight of these patients were staged T1, 13 had T2 disease and 3 had T3 disease. Eleven patients had PSA <10 ng/ml, 8 had PSA 10 – 20 ng/ml and 5 had PSA >20 ng/ml. Gleason score was ≤ 6 in 11 patients, 9 had Gleason score 7 and 4 had Gleason score ≥8.

### Imaging and treatment

Patients were treated to a total dose of 74 Gy in 37 fractions, 2 Gy per fraction, 5 days per week on a Siemens PRIMATOM (Siemens OCS, Concord, USA) linear accelerator. Patients were instructed to empty their bladder and rectum one hour before treatment and to drink 750 ml of water. They were immobilized supine on a carbon fiber couch top with foot stocks and knee support. Initial setup was by alignment of three skin tattoos to lasers. Setup was verified by a pre-treatment CT scan prior to each fraction (from a Siemens SOMATOM diagnostic CT scanner, using the same couch top and mounted on rails). CT slices were reconstructed with 5 mm thickness and spacing. If the rectal diameter was over 4 cm on the pre-treatment CT, the patient was taken off the bed and asked to empty their rectum before re-CT and treatment. The first pre-treatment CT for each patient was used as the reference image for movement analysis as this was closer in time to the rest of treatment than the planning scan.

### Volume delineation

CT on rail images were imported into XiO^TM^ CMS (Elekta Ltd, Crawley, UK). Prostate, bladder and rectum were contoured by a single investigator (KK). Two investigators (DM, AS) contoured SV, with all the images in one patient study set contoured by the same investigator. At the start of the study both investigators contoured test cases and compared results to ensure their work was consistent.

### Displacement analysis

The contours were exported as DICOM structure sets. Software was written in C++ using the dicomlib toolkit (available at http://code.google.com/p/dicomlib/) to read the structure sets and calculate volumes and centroid positions using the original contours. Centres of volumes (centroids) were calculated for the entire SV, the inferior 2.5 cm and the tip (most superior slice) of the SV. The prostate centroid was used as the origin on each fraction, with the other centroid positions (i.e. SV movements) being reported relative to this point. Translational movement was measured, but not rotational displacement.

### Assessment of margins

SV margins were assessed using two methods (i) the van Herk formula (margin = 2.5 x systematic error + 0.7 x random error) [6], and (ii) geometrically based on isotropic expansion of SV contours. For the second method, software was written to interpolate the reference SV contours onto a 1 mm x 1 mm x 1 mm grid. The left and right SV for each patient were considered separately. All voxels inside the contours were marked to form a volume. An isotropic 3D margin was applied to this reference SV volume (CTV) using convolution for each margin size. The margin size was increased in 1 mm increments. For each margin size, the appropriate (left or right) SV contours from each daily treatment scan were overlaid onto the voxel grid, after alignment of the prostate centroids, to check if the points forming the contours would fit inside the grown reference SV volume. If any part was outside by more than 1 mm, coverage was considered as inadequate. This process was repeated with the inferior 2.5 cm of each SV considered.

### Correlation of SV movement with organ volume

In order to obtain the average rectal area and volume, two separate methods were used in this study. All volumes were calculated by the software mentioned above.

### Method 1

Method 1 was conducted to see if the volume of rectum adjacent to the SV correlated with day to day SV displacement since the rectum is more easily localized than SV on pretreatment imaging such as cone beam CT. Only the section of rectum adjacent to SV on the reference scan was used for analysis. To assess the rectal parameters during treatment, only the section of rectum in the same CT slice positions (relative to the prostate centroid) as on the reference scan was examined. Total rectal volume was measured, and area was calculated by dividing the volume by the slice width and the number of slices which contained rectum. If there was significant rectal movement during treatment, the number of CT slices containing rectum in the predefined CT slice positions could be smaller. In fact, there was significant rectal movement in two patients where there was no rectum seen in the correlating CT slices on treatment, and hence, method 2 was used to calculate rectal volume and area.

### Method 2

Rectal volume and area were measured from the level of the prostate centroid to the recto-sigmoid junction. This method will be valid for all patients, however, correlations with SV movement was expected to be weaker as a longer length of rectum was considered, where a significant portion of rectum was not adjacent to SV.

### Statistical analysis

Mean and standard deviation (SD) were used to describe the variation in volumes of relevant organs during the course of treatment. For the SV movement analysis, both systematic and random errors were calculated. Linear regression analyses were used to explore the correlation (R^2^) of SV displacements in relation to contralateral SV displacement, bladder volume, motion of the most posterior point of the bladder, rectal area and rectal volume.

## Results

### Random and systematic error for SV displacements

Systematic and random displacement of SV centroids and margins from the van Herk formula are given in Table 2. Differences between LSV and RSV were not statistically significant.

**Table 2:** Systematic and random errors for displacement of the three points of interest for the right seminal vesicle (RSV) and left seminal vesicle (LSV)

	**Direction**	**Whole LSV**	Whole LSV* (long)	Inferior 2.5 cm LSV^*^	**LSV tip**	**Whole RSV**	Whole RSV* (long)	Inferior 2.5 cm RSV^*^	**RSV tip**	**Whole SV†**	**Inferior 2.5 cm SV†**	**SV tip†**
Systematic error (mm) (∑)	LR	1.5	1.3	1.3	3.3	2.1	1.6	1.7	4.4	1.9	1.9	4.2
	AP	2.7	3.1	2.7	3.9	3.0	3.2	3.6	4.2	2.9	2.9	4.0
	SI	3.1	3.4	3.5	4.0	4.1	3.3	3.1	5.8	3.6	3.5	5.0
Random error (mm) (σ)	LR	1.3	1.3	1.3	3.3	1.5	1.6	1.5	3.9	1.4	1.4	3.6
	AP	2.7	2.9	2.9	4.0	2.7	2.7	2.8	4.1	2.7	2.8	4.1
	SI	2.1	2.1	2.1	3.5	2.1	2.3	2.2	3.5	2.1	2.1	3.5
van Herk Margin (mm)	LR	4.7	4.2	4.2		6.3	5.1	5.3		5.7	5.7	
	AP	8.6	9.8	8.8		9.4	9.9	11.0		9.1	9.2	
	SI	9.2	10.0	10.2		11.7	9.9	9.3		10.5	10.2	

*Data from 13 LSV or 15 RSV, as the remaining SVs were shorter than 2.5 cm on the reference scan.

†Combined data for both SVs, all patients.

### Geometric margin based on isotropic SV expansion

**Figure 1:**
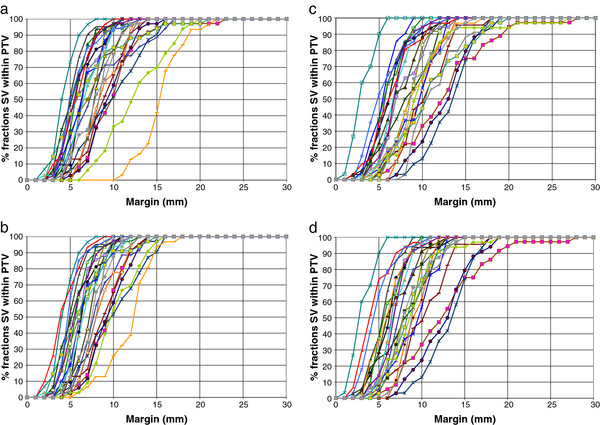
** a) Margins required to cover the entire left seminal vesicle.** Results for all 24 patients were plotted individually. b) Margins required to cover the inferior 2.5 cm portion of left seminal vesicle, c) entire right seminal vesicle and d) inferior 2.5 cm portion of right seminal vesicle. Same line colour has been used for each individual patients in Figures 1a – d.

**Figure 2:**
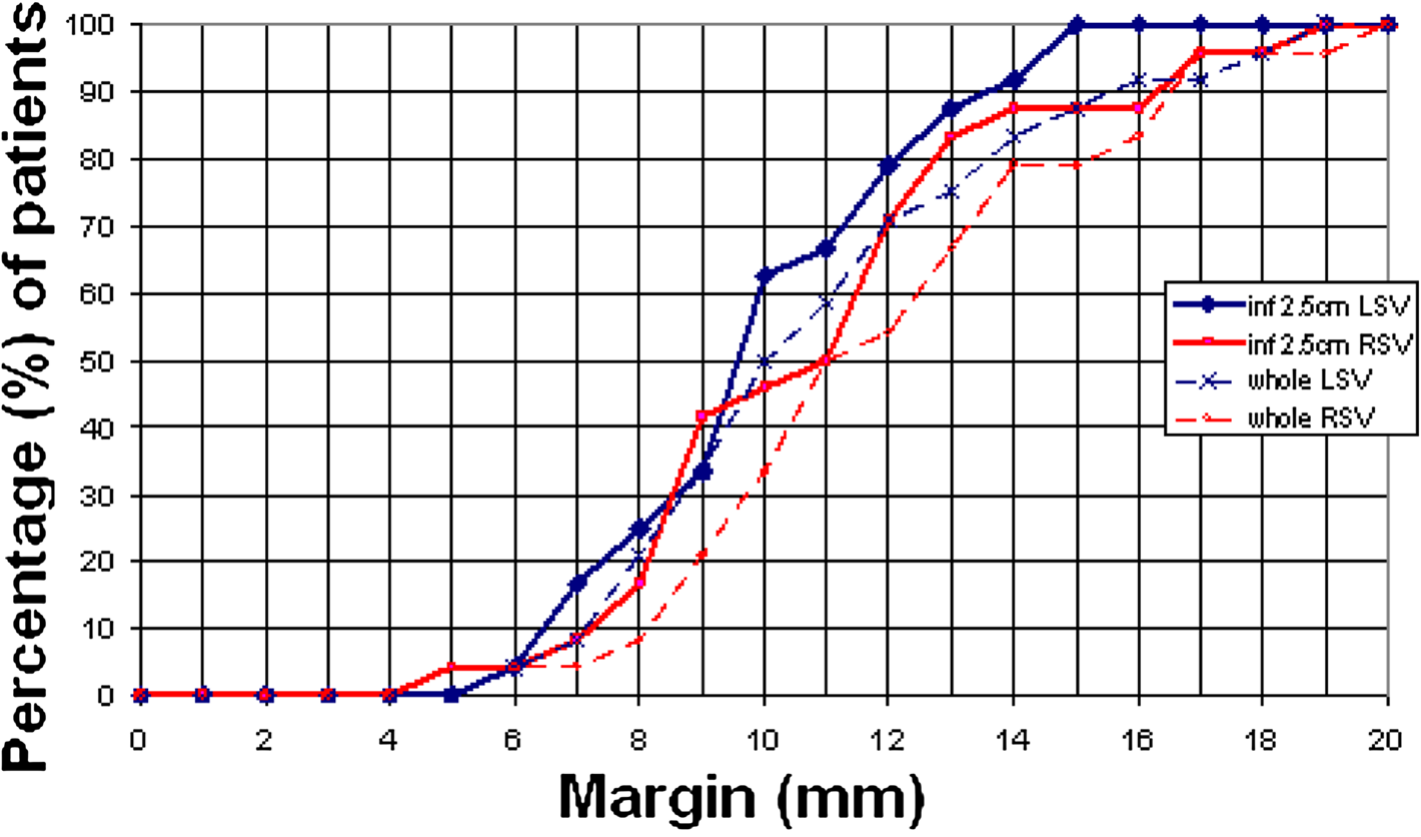
** Percentage of patients where the entire or the inferior 2.5 cm of SV is within the PTV on at least 90% of the fractions.** Data from all patients including those with SVs ≤ 2.5 cm in length.

A margin of 15.6 mm was required to cover the whole left SV for 90% of fractions for 90% of the entire patient cohort. For the right SV, a margin of 16.5 mm was required. For the inferior 2.5 cm of left and right SV, margins of 13.6 mm and 16.3 mm were required. (Figures 1, Figures 2). The mean margins required for each patient to cover 90% of the fractions were as follow: entire LSV 11.3 mm (SD 3.4 mm), inferior 2.5 cm LSV 10.3 mm (SD 2.5 mm), entire RSV 12.3 mm (SD 3.5 mm), and inferior 2.5 cm RSV 11.2 mm (SD 3.3 mm).

There were 13 patients with LSV and 15 patients with RSV which were longer than 2.5 cm on the reference scan. In this subgroup, the mean margins for each patient to cover 90% of their fractions were: entire LSV 12.1 mm (SD 3.6 mm), inferior 2.5 cm LSV 10.8 mm (SD 2.4 mm), entire RSV 12.0 mm (SD 2.8 mm), and inferior 2.5 cm RSV 11.0 mm (SD 2.9 mm).

**Figure 3:**
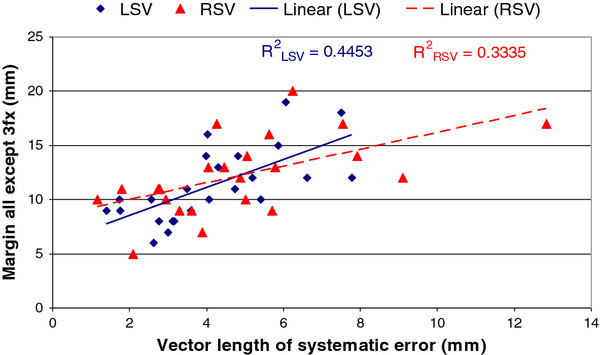
** Relationship between the geometrical margin to cover SV and systematic error (average movement of the seminal vesicle centroid from its position in the reference scan, expressed as the length of the 3D vector).** Three fractions were excluded as outliers which give margins that fit approximately 90% of all fractions.

Figures 3 demonstrates the relationship between geometrical margins and systematic error (the amount by which the position of the SV centroid on the reference scan, relative to the prostate centroid, was not typical for the rest of the treatment, for example due to rectal gas in the reference scan.)

### Variation in volumes

The mean superior-inferior (SI) length of SV on all CT images was 2.7 cm (range 1 to 4.5 cm with CT slice spacing of 0.5 cm) for the left SV and 2.8 cm (range 1 to 5 cm) for the right SV. In those patients (13 LSV, 15 RSV) where LSV or RSV was longer than 2.5 cm on the reference scan, the mean length on the reference scan was 3.3 cm for both LSV and RSV. Mean left SV, right SV and prostate volumes on the reference scan were 8.3 cm^3^, 8.8 cm^3^ and 32 cm^3^ respectively. The SD of volume variations during treatment for LSV was 1.28 cm^3^, RSV 1.22 cm^3^, and prostate 4.58 cm^3^.

The mean bladder volume of all reference scans was 258 cm^3^. After some smoothing of day to day volume variation (median SD 62 cm^3^), three patients had a significant trend of bladder volume as treatment progressed (R^2^ > 0.2). For these three patients, bladder volume was reduced by 1.2%, 1.4% and 0.9% per fraction on average.

The mean rectal area (method 1) of all reference scans was 14.1 cm^2^ with a median day to day variation (SD) of 3.7 cm^2^. After smoothing, four patients had a significant trend as treatment progressed (defined as R^2^ > 0.2 and change per day greater than 0.15 cm^2^). The rectal area for these four patients was reduced by 1.0%, 1.9%, 1.7% and 2.0% per fraction on average respectively.

### SV displacement in relation to bladder, rectum and contralateral SV

Correlation of contralateral SV movements was seen in the SI (R^2^ = 0.72) and anterior-posterior (AP) (R^2^ = 0.44) directions, but not in the left-right (LR) direction.

**Figure 4:**
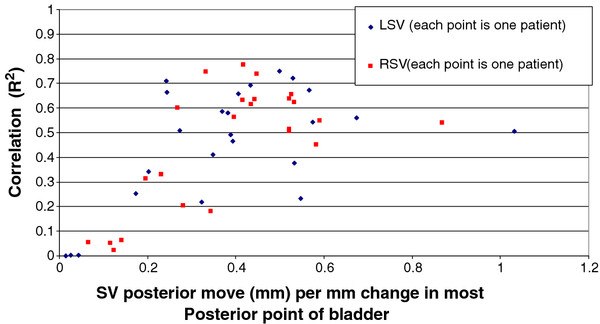
Correlation of SV AP movement with movement of the most posterior point of the bladder.

There was a weak association between AP SV motion and bladder volume. Out of twenty four, six left SV and seven right SV had a correlation (R^2^) ≥ 0.2. However, a strong correlation was seen between AP movement of SV and the movement of the most posterior point of the bladder as shown in Figures 4. On a per patient basis the mean correlation (R^2^) for the left SV was 0.46 with a SD of 0.23. For the right SV, the mean correlation (R^2^) was 0.46 with a SD of 0.24.

**Figure 5:**
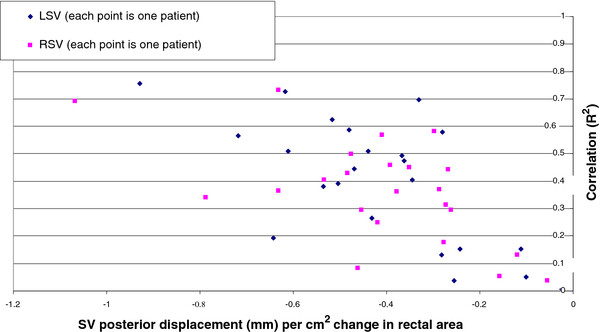
** Correlation of SV AP movement with change in rectal area using Method 1 of rectal area evaluation.** Data for both LSV and RSV for all patients were plotted individually. A negative value on the x axis represents anterior movement of SV as rectal area increases.

Correlation between SV movement and rectal area and volume is shown in Figures 5 and Table 3. Weak correlations (mean R^2^ ~ 0.2) were observed in the SI and LR directions. Stronger correlations (mean R^2^ up to 0.4) were observed in the AP direction with method 1 showing slightly stronger correlations than method 2. Rectal area showed a slightly greater correlation than rectal volume with AP SV movement. Method 1 could not be used for 28/31 fractions of one patient, and 2/34 fractions of a second patient, due to the recto-sigmoid junction moving to a position inferior to SV on the reference scan.

**Table 3:** Rectal area and volume correlations with SV movements

		Rectal contour using Method 1*				Rectal contour using Method 2*			
		**Rectal area**		**Rectal volume**		**Rectal area**		**Rectal volume**	
		**Average correlations (R**^**2**^**)**	**SD**	**Average correlations (R**^**2**^**)**	**SD**	**Average correlations (R**^**2**^**)**	**SD**	**Average correlations (R**^**2**^**)**	**SD**
Ant/Post	LSV	0.40	0.23	0.37	0.22	0.38	0.24	0.35	0.21
	RSV	0.36	0.19	0.35	0.16	0.35	0.21	0.33	0.17
Sup/Inf	LSV	0.20	0.17	0.22	0.19	0.21	0.18	0.23	0.19
	RSV	0.22	0.17	0.23	0.19	0.20	0.16	0.24	0.20
Left/Right	LSV	0.19	0.19	0.19	0.19	0.21	0.20	0.17	0.18
	RSV	0.18	0.18	0.15	0.18	0.20	0.18	0.15	0.17

* Rectal contouring methods have been described in the Methods and Materials section.

## Discussion

This study assessed a large number of scans (median 32 per patient) for a considerable number of patients (24) and found considerable SV motion relative to the prostate. We looked at a sufficient number of patients to see some with large systematic errors requiring larger margins. Assessing a large number of pre-treatment CTs enabled us to use actual SV contours for the movement at each fraction, rather than assuming a normal distribution.

Table 1 gives an overview of the studies looking at SV movements and margins required for SV in radiotherapy. The papers using a dose-based analysis generally find considerably smaller margins, as do those when the prostate and SV were assessed in combination. Studies using prostate fiducial markers or the prostate centroid for localization report reasonably similar movements.

We estimated margins in two ways, using the van Herk formula, and by considering geometric coverage using an isotropic expansion from the reference CT. The van Herk formula estimates the probability of distribution of the cumulative dose over a population of patients, but excludes rotational errors and shape deviations. Our geometric analysis allows for rotation and deformation but ignores dose outside of the PTV. Both methods reported large margins, with the van Herk formula estimates closely approximating findings from other studies [7,8]. Larger margins of 15.6 mm and 16.5 mm were required to cover the whole left and right SV for 90% of fractions for 90% of the entire patient cohort in this study using the geometrical analysis. These resultant margins are not recommended for treatment purposes given the potential toxicities from dose to bladder and rectum. Moreover, small excursions of SV outside of the PTV have only a limited effect on the cumulative SV dose, particularly if different parts of SV are missed on different treatment fractions. Dose outside of the PTV is likely to be higher for the SV because dose “fall off” from the superior part of the prostate will contribute to dose to the SV. In addition, there are some limitations of our study. Although mitigated by the use of interpolation, our geometric method is limited by the 5 mm thick CT slice spacing, with the greatest limitation in the estimation of SI margin. As a result, reported movements of the SV tip will be less accurate than movements of the whole SV and inferior 2.5 cm portion. Uncertainty in manual contouring of SV on CT slices will have led to a slight increase in reported movement and margins. Combining these effects, margin for SV is likely to be much smaller than the geometrical margins reported here. Nevertheless, our data show that movement of SV relative to the prostate is substantial and should be considered when setting margins for conformal treatment techniques where daily setup is to the prostate, particularly as the dose outside the PTV is likely to be reduced as radiotherapy technology improves.

This study found that the margin required to cover the proximal 2.5 cm was only a little smaller than the margin required to cover the entire SV. Differences of up to 4 mm in the margin to treat 90% of fractions could be seen for a few patients but the average difference was 1.2 mm. One might expect that displacement and hence margins were greater as distance increased from the prostate centroid. However, in those patients where the SV were longer than 2.5 cm, it was frequently not very much larger (mean 3.3 cm for both LSV and RSV). This may explain why the difference in margin for the entire SV and proximal 2.5 cm were similar.

The extent of SV inclusion is a clinical judgment often individualized to the patient depending on the proximity of rectum and small bowel. Guidelines state however that either the proximal half [1], the proximal 1–2 cm [9] or at least the proximal 1 cm of SV should be included in the high risk CTV and treated to full dose [10]. A frequently quoted study from the William Beaumont Hospital looked at 81 positive SV from clinically staged ≤ T2c prostate cancer patients who subsequently underwent prostatectomy [11]. The pattern of SV invasion was contiguous in 86%. Of those SV that were positive, 47% had SV involvement beyond the proximal 25% of the SV, 14% had SV involvement beyond the proximal 50%, and 2% were involved beyond the proximal 60%. However, the question of length of SV requiring inclusion in the radiotherapy CTV is still incompletely answered. In addition, dose required to treat microscopic disease may be reduced. Further research is required.

Even with a standardized departmental setup protocol, we have noted that there can be significant changes in bladder volume and rectal filling during the course of radiotherapy for some patients. Bladder volumes in our study did not have a good correlation with SV displacement, but a strong correlation of SV AP movement with the position of the most posterior point of the bladder was observed. Correlations with rectal filling were also observed in our study, as by Frank *et al.*[7]. Therefore, patient education and monitoring of bladder and rectal parameters are encouraged to detect both random and particularly systematic movements. Systematic and random errors might also be reduced by the use of an endorectal balloon prior to each treatment to provide constant rectal volume [12].

In a study from the William Beaumont hospital, adaptive margins for prostate and SV were created using a bounding volume of daily pre-treatment imaging in the first week, or two weeks, of treatment which improved efficacy [13]. Supporting this, our data (Figures 1, Figures 3) show that for many patients margin size is reasonably consistent between fractions and has a component related to systematic error. Given the non-uniform SV movement as noted in this study, asymmetric margins could be explored in future studies with a smaller margin in the left-right direction.

## Conclusion

Considerable interfraction SV displacement was observed in this cohort of patients with simulated IGRT targeting the prostate. Future radiotherapy technique design should not neglect SV motion. Correlation of SV motion with bladder and rectal parameters is observed.

## Competing interests

The authors declare that they have no competing interest.

## Authors’ contribution

All authors read and approved the final manuscript.
